# End-to-end dialogue structure parsing on multi-floor dialogue based on multi-task learning

**DOI:** 10.3389/frobt.2023.949600

**Published:** 2023-05-03

**Authors:** Seiya Kawano, Koichiro Yoshino, David Traum, Satoshi Nakamura

**Affiliations:** ^1^ Guardian Robot Project, RIKEN, Kyoto, Japan; ^2^ Graduate School of Science and Technology, Nara Institute of Science and Technology, Nara, Japan; ^3^ USC Institute for Creative Technologies, Los Angeles, CA, United States

**Keywords:** multi-floor dialogue, natural language understanding, dialogue structure parsing, dialogue system, human-robot dialogue

## Abstract

A multi-floor dialogue consists of multiple sets of dialogue participants, each conversing within their own floor. In the multi-floor dialogue, at least one multi-communicating member who is a participant of multiple floors and coordinates each to achieve a shared dialogue goal. The structure of such dialogues can be complex, involving intentional structure and relations that are within or across floors. In this study, We proposed a neural dialogue structure parser with an attention mechanism that applies multi-task learning to automatically identify the dialogue structure of multi-floor dialogues in a collaborative robot navigation domain. Furthermore, we propose to use dialogue response prediction as an auxiliary objective of the multi-floor dialogue structure parser to enhance the consistency of the multi-floor dialogue structure parsing. Our experimental results show that our proposed model improved the dialogue structure parsing performance more than conventional models in multi-floor dialogue.

## 1 Introduction

Recent advances in computer science make it possible to build interactive robots in some defined social roles. When such robots operate in our living space, they need to understand the conversations on multiple conversational units and the relationships between them. The physical or psychological space in which these conversations occur is often referred to as *floor*. For example, a waiter in a restaurant goes around between the kitchen and the customer seats, different floors, to understand what is going on each floor and to communicate with participants on each floor. If we build a waiter robot, the robot would need to understand how the dialogue on each floor, e.g., the content of an order, affects the dialogue on the different floors, e.g., checking the availability of the ordered food items. Parsing dialogue structure on multi-floor dialogue is necessary to realize such a robot.

Formally, the floor is defined as the acknowledged what’s-going-on within a psychological time/space (Edelsky, 1981). What’s-going-on on can be the development of a topic or a function (teasing, soliciting a response, etc.) or an interaction of the two. It can be developed or controlled by one participant at a time or by several simultaneously or in quick succession. A dialogue between two or more participants is usually conducted on a single floor, which is called a *single-floor dialogue*. By contrast, when two or more floors exist in parallel, a complex dialogue structure becomes apparent (Cherny et al., 1999). For example, although an internet relay chat (IRC) has a single message stream, multiple participants might be simultaneously chatting on different topics. At a cocktail party, people organize several groups in the same physical space and chat with each other within each group (Aoki et al., 2003; Aoki et al., 2006). An individual participant could be involved in all dialogue floors in such cases because the dialogue contents are freely visible to all participants.

**FIGURE 1:**
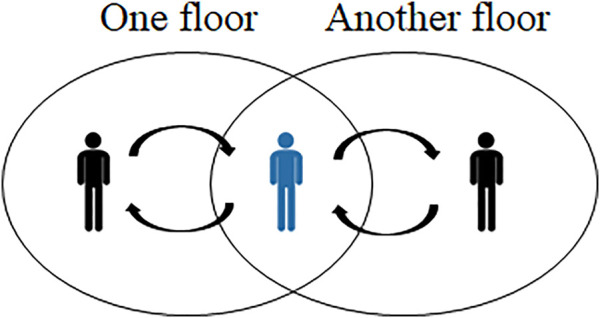
Illustration of a minimum architecture of multi-floor dialogue: one multi-communicating member (highlighted in blue) mediates the communication between two floors, including two participants.

In this study, we are interested in a more practical case called a *multi-floor dialogue*, which consists of multiple sets of dialogue participants, each conversing within their own floor, but also at least one multi-communicating member (but not all) who is a participant of multiple floors and coordinating each to achieve a shared dialogue goal (Traum et al., 2018). In the example of waiter robot, the robot communicates with customers to take their orders in the dining room (one floor) and talks with other workers in the kitchen (another floor) who prepare the customer’s food. All the participants work toward the joint goal of providing the customer with their desired meals. Another example is in military units, where soldiers follow their commander’s orders, which are decided at headquarters. Such situations are quite common in the real world, where we have different dialogue floors for decision-making and actions based on decisions (Figure 1).

Identifying aspects of multi-floor dialogue structure can be critical for building cooperative applications that have to participate in multi-floor dialogues, for example, collaborative navigation robots (Bonial et al., 2018; Lukin et al., 2018). However, most existing studies on dialogue structure parsing addressed only single-floor dialogues. There are standard annotation schemes for both dialogue acts (Bunt et al., 2012) and discourse relations (Prasad and Bunt, 2015) in single-floor dialogues. However, these schemes do not fully address the issues of dialogue structure in multi-floor dialogues. Previous work proposed an annotation scheme of dialogue structure on multi-floor dialogues (Traum et al., 2018). This scheme is based on two important aspects of dialogue structure: transaction units and the relations between utterances. A transaction unit clusters utterances from multiple participants and floors that contribute to achieving the initiating participant’s intention. Relations link utterances to antecedents within the unit. We can view parsing the dialogue structure on multi-floor dialogue as a problem that extracts directed graphs corresponding to a span of transaction units within the dialogue. However, there is still a lack of previous work on automatic dialogue structure parsing for multi-floor dialogue (Kawano et al., 2021).

In this study, we propose a dialogue structure parser for parsing multi-floor dialogue structure based on an annotation scheme of multi-floor dialogue (Traum et al., 2018). Our proposed parser aims to achieve consistent parsing of the dialogue structure by jointly handling these aspects of the multi-floor dialogue using multi-task learning. Our proposed parser is based on a two-stage cascade architecture based on deep recurrent neural networks with an attention mechanism. In the first stage, our parser resolves the link relations in each utterance and the processing status of transactions based on a supervised attention mechanism. In the second stage, our parser predicts each link’s relation type as attributes. Furthermore, our proposed parser learns the dialogue response generation task based on the resolved dialogue structure as an auxiliary task to enhance the consistency of the resolved dialogue structure. We clarify a property of the dialogue structure parsing task of multi-floor dialogue and the effectiveness and limitations of the proposed parser through a comparison between the proposed parser and the baseline model trained under various settings.

In the following sections, we first describe the previous work of dialogue structure parsing (Section 2). Then, we describe the dialogue structure parsing task on multi-floor dialogue, an annotation scheme, and our target domain (Section 3; Section 4). We describe our proposed parser based on the deep neural network, which has cascade architecture (Section 5). We experimentally evaluated our model’s dialogue structure parsing performance under training on different kinds of settings, using automatic metrics that focus on micro- and meso-level structures (Traum and Nakatani, 1999) in dialogues (Section 6). Our proposed model using multi-task learning improved the overall parsing performance of dialogue structures compared to other baseline models (Section 7). Finally, we summarize the key conclusions of this work and discuss some future directions of this work (Section 8).

## 2 Related work

The major research on dialogue structure parsing can be broadly categorized into identifying micro- and macro-structures in dialogue. In the former, the intentions of individual utterances in a dialogue are automatically identified. Dialogue act labels (Jurafsky, 1997; Stolcke et al., 2000; Kawano et al., 2019) are widely used to identify the intentions of utterances, and the task of assigning dialogue act labels to utterances is known as dialogue act classification or dialogue act recognition. The latter reveals the process of communicating intentions to achieve the goal of the dialogue. The dialogue state tracking task sequentially estimates the relevant information needed to achieve the dialogue goal based on the representation represented by slot-value pairs as the dialogue progresses (Williams et al., 2016; Mrkšić et al., 2017) In addition, discourse relation analysis identifies the dependency relations of each discourse unit or utterance in dialogue, which constitutes the sentence to be analyzed (Ji and Smith, 2017; Shi and Huang, 2019). However, most of these existing studies on dialogue structure parsing deal only with single-floor dialogues and do not fully address the issue of dialogue structure in multi-floor dialogues.

Some studies have focused on conversation situations where multiple floors are in parallel. For example, in Internet Relay Chat (IRC) and communication platforms such as slack, conversations on different topics often exist in parallel on a single message stream. In addition, at cocktail parties, parallel conversations are held by multiple small groups with a fluid structure of participants. Techniques for separating these entangled conversations into separate conversations are known as conversation disentanglement (Elsner and Charniak, 2008; Kummerfeld et al., 2019; Yu and Joty, 2020; Liu et al., 2021). Conversation disentanglement is important for analyzing conversations within each floor of entangled conversations and acquiring training resources for downstream applications such as dialogue systems.

One solution for resolving an entangled conversation is to formulate it as a topic-tracking task, such that each message in an entangled conversation determines whether it starts a new conversation or belongs to an existing conversation (Wang et al., 2020). Another solution is to identify the referential and non-referential relationships within two utterances that determine whether an utterance is a response to another utterance (Kawano et al., 2021). Supervised and unsupervised conversation disentanglement methods have been proposed for both of these approaches, and in particular, the development of neural conversation disentanglement models based on the powerful performance of deep neural networks is noteworthy (Kummerfeld et al., 2019; Yu and Joty, 2020; Liu et al., 2021). However, the main purpose of all these approaches is to extract multi-party conversations on independent topics from entangled conversations, and they do not shed light on the relationships between utterances or the information exchange process between floors. In addition, they do not address multi-floor dialogues, where only certain participants have communication channels across floors. Specifically, multi-floor dialogue parsing requires consideration of multiple floors and transaction units, and the multiple transaction units present in the dialogue have a time-series dependency. Thus, parsing of multi-floor dialogues is inherently different from typical multi-party dialogue parsing tasks that do not take such aspects into account (e.g., the conversation disentanglement task).

In this study, we investigate a framework for a robust neural dialogue structure parsing model to identify relationships between utterances or the information exchange process between floors, on multi-floor dialogues. In the following section, we describe the annotation schema of the task domain and multi-floor dialogue structure that we are targeting.

## 3 Dialogue structure in multi-floor dialogue

For our initial investigations, we use a dataset of multi-floor dialogue structure, created as part of a long-term project to develop an autonomous robot (Marge et al., 2016; Lukin et al., 2018; Gervits et al., 2019), which is commanded by remote human participants. The dataset consists of “Wizard of Oz” dialogues where two wizards control the robot and communicate with the human commander. The robot is in an unfamiliar physical environment, where it performs object searches through natural language interaction. The dialogue manager wizard (DM) communicates directly with the commander in natural language and handles clarifications or misconceptions that might not be applicable given the environment and robot capabilities. A robot navigator wizard (RN) controls the robot with a joystick controller, but communicates only with the DM. There are thus two separate floors - one between commander and “robot” (actually the DM), and one between the two wizards. These floors are called “left” and “right”, for convenience. The dialogues included in the dataset are transcriptions of spoken dialogue, which may contain grammatical errors, ambiguity, and complex turn-taking. Table 1 displays an example of a fragment of actual dialogue that includes two floors and four distinct message streams. The commander gives its intention to the DM on their dialogue floor (left floor). The DM talks with the commander (when necessary) to clarify the commander’s intention (clarification-request). After completely understanding the commander’s intention (clarification-repair), the DM moves to another dialogue floor (right floor) to transfer the summarized commander’s intention to the RN (translate-r), which operates the robot based on the given intention and reports the result to the DM (ack-done). The DM returns to the first floor to give feedback on the result to the commander (translation-l). Note that the DM can communicate with any participants by moving among several dialogue floors to transfer the information as a multi-communicator (Reinsch Jr et al., 2008); but the RN and the commander cannot directly communicate. Therefore, the DM needs to act as an intermediary between the robot and the commander to convey their intentions to each other.

**TABLE 1:** Dialogue example of multi-floor dialogue.

	Left Floor		Right Floor		Annotations		
#	Commander	DM → Commander	DM → RN	RN	TU	Ant	Rel
1	move to where you see the first cone				1	#	#
2		I’m not sure which object you are referring to. Can you describe it in another way, using color or its location?			1	1	clarification-request
3	move to the cone on the right a red cone on the right				1	2	clarification-repair
4			move to face the cone on the right		1	3	translation-r
5		executing…			1	3	ack-doing
6	take another picture				2	#	#
7				done	1	4	ack-done
8		done			1	7	translation-l
9			image		2	6	translation-r
10				image sent	2	9	ack-done
11		sent			2	10	translation-l

Previous work defined an annotation scheme for such multi-floor dialogues to specify their characteristics (Traum et al., 2018). To capture the information update process of the dialogue participants, this scheme focused on the intentional structure (Grosz and Sidner, 1986), which consists of units of multiple consecutive utterances, and the relations between pairs of utterances within the unit. They defined an annotation scheme for 1) transaction units, 2) antecedents, and 3) relation-types, and the dataset includes human-annotated data. In this study, we explore a model that automatically identifies these structures. Below we describe the annotation scheme in (Traum et al., 2018).

### 3.1 Transaction unit

A transaction unit (TU) is a basic unit of intentional structure in a multi-floor interaction. It consists of the initial utterance that expresses the intention of the speakers and every subsequent utterance across all the floors to achieve the original speaker’s intention. Each utterance belongs to a transaction unit, which is defined by a set of utterances. The “TU” column of Table 1 shows an numerical identifier for the unit which is the same for all utterances that are part of the TU.

In some cases, multiple transactions are “active” at the same time, in that they have been initiated but not terminated. For example, Table 1 shows a case where two transaction units are included in the dialogue: TU1 is about moving somewhere, while TU2 is about taking a picture. TU2 is initiated in utterance #6, before TU1 is completed in utterance #8. Both transactions are thus running in parallel during this part of the dialogue.

### 3.2 Antecedent and relation-type

In (Traum et al., 2018), relations are annotated between utterances in the same TU, using antecedents and relation-types. All utterances after the first utterance in the transaction unit have antecedents, shown in the “Ant” column of Table 1, as the utterance ID of the antecedent utterance. Relation types are summarized in Table 2. These relations are categorized first as to whether they are from the same participant (expansions), from different participants on the same floor (responses), or across floors (translations). Each of these categories has a set of specific relations and, in some cases sub-types. Relation types are indicated in the “Rel” column in Table 1. For detailed definitions of each relation type, please refer to (Traum et al., 2018) ^1^.

**TABLE 2:** Relation-types in a multi-floor dialogue.

Type	Sub-types
Expansions	relate utterances that are produced by the same participant within the same floor
	continue
	link-next
	correction
	summarization
Responses	relate utterances by different participants within the same floor
	acknowledgment
	done
	doing
	wilco
	understand
	try
	unsure
	cannot
	clarification
	req-clar
	clar-repair
	missing info
	nack
	repeat
	processing
	question-response
	answer
	non-answer
	other
	3rd turn feedback
	reciprocal response
Translations	relate utterances in different floors
	transalation-l
	transalation-r
	comment
	quotation

The set of relations within a transaction define a graph structure, where the first utterance has no relation-type or antecedent. In the example in Table 1, #1 and #6 are the first utterances of the two transaction units. Here, the hash symbol (#) is used to indicate that no antecedent and relation-type are assigned to these utterances.

## 4 Problem formulation

**FIGURE 2:**
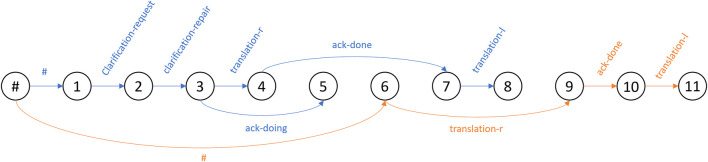
Graphical example of multi-floor graph structure corresponding to dialogue example in Table 1.

We first formulate the problem of parsing dialogue structure on a multi-floor dialogue. The problem is to extract multiple subgraphs from a dialogue session, each of which has a relation-type as an edge attribute, corresponding to transaction units within the dialogue session. Specifically, an entangled multi-floor dialogue can be represented as a directed graph *G* (*V*, *E*). Here, *V* is a set of *m* nodes {1, … , *m*}. $E={\left\{e_{i,j}\right\}}_{i,j=1}^{m}$ is a set of *m* directed edges with attributes, where *i* and *j* are nodes of the graph, which in our problem setting means an utterance. If there is a dependency relation from the utterance *i* to *j*, we represent *e*_*i*,*j*_ as one-hot vector representing corresponding relation-type of edge. In order to integrate multiple isolated graphs corresponding to transaction units into one graph, we introduced a root node as a shared pseudo-antecedent utterance to the starting utterance of a transaction unit. In other words, one dialogue is represented by a graph that headed by the root node, and the goal of dialogue structure parsing is to output a graph structure of a given dialogue. We can define two settings for inducing the graph structure of a given dialogue: online and offline. The online setting assumes incremental inputs, while the offline setting assumes a batched input. A graphical example of the multi-floor dialogue in Table 1 is shown in Figure 2. Here, the numbers in the nodes are the indices of utterances, where # indicates the root node. Blue links indicate the case of TU1, and orange links indicate the case of TU2.

## 5 Neural dialogue structure parser for multi-floor dialogue

In this section, we introduce a neural dialogue structure parser for the annotation scheme proposed by (Traum et al., 2018). We can view parsing the dialogue structure on multi-floor dialogue as a problem, which extracts directed graphs corresponding to a span of transaction units within the dialogue.

Our dialogue structure parser uses a two-stage cascade structure, consisting of a dialogue disentanglement model that disentangles entangled multi-floor dialogues and predicts the relation-types and transaction boundaries of two utterances based on the dependency relations between the entangled utterances. The model leverages the fact that the definitions of transaction units, antecedents, and relation types that make up a multi-floor dialogue are closely related. It was intended to encourage the overall performance of dialogue structure parsing by simultaneously solving them based on multi-task learning. The front-end network of the model, the attention mechanism that resolves link relations between nodes, is inspired by a powerful baseline model of discourse structure parsing and dependency parsing (Zhang et al., 2017; Shi and Huang, 2019). It has much in common with our dialogue structure parsing task in that it extracts graph structure from the text. However, these models do not fully address the issues of dialogue structure in multi-floor dialogues. Specifically, multi-floor dialogue parsing requires consideration of multiple floors and transaction units, and the multiple transaction units present in the dialogue have a time-series dependency. Thus, parsing of multi-floor dialogues is inherently different from typical multiparty dialogue parsing tasks that do not take such aspects into account (e.g., conversation disentanglement task). In this study, we generalize and extend previous works (Zhang et al., 2017; Shi and Huang, 2019; Kawano et al., 2021) to fit the problem of multi-floor dialogue structure parsing. Additionally, we propose an auxiliary objective function to enhance the consistency of the predicted multi-floor dialogue structure.

**FIGURE 3:**
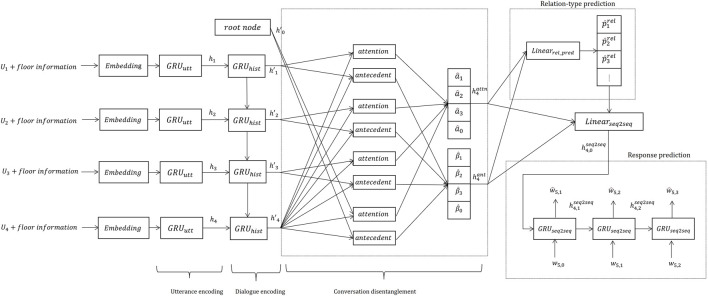
Overview of proposed neural multi-floor dialogue structure parser (prediction case at *t* = 4).

Our proposed model (Figure 3) mainly includes four networks:

• The hierarchical encoding layer has utterance and context encoders for encoding each dialogue context in different dialogue levels.
• The conversation disentanglement layer estimates the antecedent corresponding to each utterance. The layer also uses the root node as an antecedent candidate. If the root node is selected, it means that the utterance is a beginning of a new transaction unit.
• The relation-type prediction layer estimates the relation-type of each utterance and its antecedent.
• The response prediction layer generates the expected precedent response corresponding to each utterance using the predicted dialogue structure.

The relation-type predictors share the prediction results of the conversation disentanglement with other layers as attention weights, because their prediction results are related to the potential graph structures decided by the antecedent predictor model. Such a two-stage approach, which predicts the dependency structure of the utterances and its relation-types, resembles previous work (Shi and Huang, 2019). However, that model targets single-floor dialogue structure parsing, and our model predicts the dialogue structure of multi-floor dialogues and clusters the utterances in different floors as one transaction unit. Additionally, we are introducing a novel response prediction layer that employs disentangled dialogue history (dialogue structure) to predict responses. This objective is based on the heuristic that a general dialogue response generation model, i.e., a dialogue system, should be able to predict the correct response when the given dialogue context is disentangled, but it will be perplexed when the context is entangled. By disentangling the dialogue, we can accurately predict the correct response, and the objective function of the response generation model (response prediction layer) can provide feedback on what the better dialogue structure is. We expect that the response generation objective can contribute to the dialogue structure parsing because dialogue disentanglement is useful for predicting the appropriate response. We formally define each layer as follows.

### 5.1 Hierarchical encoding layer

Our hierarchical encoder consists of utterance and context encoders based on recurrent neural networks (RNNs). ^2^The utterance encoder receives a word at each time step using forward and backward GRUs (Cho et al., 2014) to encode each utterance into a fixed-length vector:

$$\vec{h_{t,i}}={\vec{\text{GRU}}}_{\text{utt}}\left(\vec{h_{t,i-1}},\text{Embedding}\left(w_{t,i}\right)\right),$$ (1)

$$\overset{⃖}{h_{t,i}}={\overset{⃖}{\text{GRU}}}_{\text{utt}}\left(\overset{⃖}{h_{t,i+1}},\text{Embedding}\left(w_{t,i}\right)\right),$$ (2)

$$h_{t,i}=\left[\vec{h_{t,i}};\overset{⃖}{h_{t,i}}\right],$$ (3)

$$h_{t}=\frac{1}{\left|U_{t}\right|}\overset{\left|U_{t}\right|}{\underset{i=1}{\sum }}h_{t,i}.$$ (4)

Here *t* is the utterance numbers in the dialogue context and *i* is the word order in the utterance. *h*_*t*,*i*_ is the hidden vector calculated from each word *w*_*t*,*i*_ and the hidden vector in previous time-step *h*_*t*,*i*−1_ in utterance *U*_*t*_ = [*w*_*t*,1_, *w*_*t*,2_, … , *w*_*t*,*N*_]. Each word *w*_*t*,*i*_ is converted to a fixed-length vector using an embedding layer before calculating the hidden vector. In each utterance, we added a special symbol, which indicates the types of floors, to prefixes and suffixes of utterance and trained the embedding rule as done with words.

In the context encoder, utterance vectors are input to encode the dialogue history to get context-level vector representation $h_{t}^{\prime }$ for each utterance in the dialogue contexts:

$$h_{t}^{\prime }={\vec{\text{GRU}}}_{\text{hist}}\left.\left(h_{t-1}^{\prime },h_{t}\right)\right).$$ (5)

Here, we can also use a bidirectional GRUs instead of the unidirectional GRUs if all utterances are possible from each turn to the end of the dialogue:

$$\vec{h_{t}^{\prime }}={\vec{\text{GRU}}}_{\text{hist}}\left(\vec{h_{t-1}^{\prime }},h_{t}\right),$$ (6)

$$\overset{⃖}{h_{t}^{\prime }}={\overset{⃖}{\text{GRU}}}_{\text{hist}}\left(\vec{h_{t+1}^{\prime }},h_{t}\right),$$ (7)

$$h_{t}^{\prime }=\left[\vec{h_{t}^{\prime }};\overset{⃖}{h_{t}^{\prime }}\right].$$ (8)

The bidirectional-based model (offline model)has the advantage of being able to access information from the entire dialogue, and is probably suited for situations where progressive parsing is not necessary. On the other hand, the unidirectional-based model (online model) can be useful in applications that require incremental information processing, such as dialogue systems.

We introduce a implicit-attention mechanism (Luong et al., 2015) for dialogue contexts to compute contextual representation ${\bar{h}}_{t}^{\text{attn}}$ for each utterance *U*_*t*_:

$$\text{attention}\left(h_{t-i}^{\prime },h_{t}^{\prime }\right)=h_{t-i}^{\prime T}W_{\text{ant}}h_{t}^{\prime },$$ (9)

$$\alpha_{i}=\frac{\exp\left(\text{attention}\left(h_{t-i}^{\prime },h_{t}^{\prime }\right)\right)}{\sum_{j=1}^{k}\exp\left(\text{attention}\right.\left(h_{t-j}^{\prime },h_{t}^{\prime }\right)},$$ (10)

$${\bar{h}}_{t}^{\text{attn}}=\overset{k}{\underset{i=1}{\sum }}\alpha_{i}\cdot h_{t-i}^{\prime }.$$ (11)

Here *k* is the number of previous utterances considered in the calculation of attention, *W*_attn_ is a trainable weight-matrix, and *α*_*i*_ ∈ [0,1]^*k*^.

In addition, we introduce a supervised-attention mechanism for explicitly considering the antecedent, which corresponds to each turn *t*:

$${\bar{h}}_{t}^{\text{ant}}=\overset{k}{\underset{i=1}{\sum }}{\hat{\beta }}_{i}\cdot h_{t-i}^{\prime }$$ (12)

Here *β*_*j*_ takes 1 if utterance *U*_*t*−*i*_ is the antecedent of utterance *U*_*t*_ and 0 in other cases (*β*_*i*_ ∈ {0,1}^*k*^).

Attention vectors ${\bar{h}}_{t}^{\text{attn}}$ and ${\bar{h}}_{t}^{\text{ant}}$ , which are calculated on the basis of the supervised- and implicit-attention mechanisms, are combined:

$${\hat{h}}_{t}^{\text{fc}}=\text{tanh}{\left(\text{Linear}\right.}_{\text{attn}}\left(\left[{\bar{h}}_{t}^{\text{attn}};{\bar{h}}_{t}^{\text{ant}};h_{t}^{\prime }\right]\right).$$ (13)

Here Linear_attn_ is a linear transformation layer, which includes a bias term. ${\hat{h}}_{t}^{\text{fc}}$ is a shared vector for predicting the transaction units and relation-types. Note that gold antecedent *β* is used in training; however, in the inference, the model uses predicted distribution of ${\bar{h}}_{t}^{\text{ant}}$ by the conversation disentanglement layer.

### 5.2 Conversation disentanglement layer

As shown in Table 1, each utterance has an annotation of the utterance ID of its antecedent in the Ant column. Furthermore, an utterance with no antecedent indicates the beginning of transaction unit. To predict the antecedents and the start of transaction units for each utterance *U*_*t*_, we calculated the scores between each utterance and the contextual utterances:

$$\text{antecedent}\left(h_{t-i}^{\prime },h_{t}^{\prime }\right)=h_{t-i}^{\prime T}W_{\text{ant}}h_{t}^{\prime },$$ (14)

$$\hat{\beta_{i}}=\frac{\exp\left(\text{antecedent}\left(h_{t-i}^{\prime },h_{t}^{\prime }\right)\right)}{\sum_{j=1}^{k}\exp\left(\text{antecedent}\right.\left(h_{t-j}^{\prime },h_{t}^{\prime }\right)}.$$ (15)

Here, *k* is the number of preceding utterances that can be the antecedent, *W*_ant_ is a trainable weight-matrix, and $\hat{\beta_{j}}\in {\left[0,1\right]}^{k}$ . By calculating the position of antecedent from the weights of attention, we can carry this knowledge forward to other predictions in the rater step: transaction-unit prediction and relation-types prediction.

We set the cross-entropy loss between predicted distribution $\hat{\beta }$ and actual antecedent label *β* as a loss function that enforces that the contextual utterance has the highest score when it is the antecedent of *U*_*t*_:

$$L_{t,\text{ant}}=-\overset{k}{\underset{i=1}{\sum }}\beta_{i}\log\left(\hat{\beta_{i}}\right).$$ (16)

Note that we also calculate the attention weight corresponding to the case where the utterance does not have any antecedent (#) using the trainable (dummy) vector $h_{0}^{\prime }$ and the hidden vector $h_{t}^{\prime }$ .

### 5.3 Relation-type prediction layer

We used ${\hat{h}}_{t}^{\text{fc}}$ as well as the transaction-unit predictor to predict the relation-type of each utterance with its antecedent:

$${\hat{p}}_{t}^{\text{rel}}=\text{softmax}\left({\text{Linear}}_{\text{rel\_pred}}\left({\hat{h}}_{t}^{\text{fc}}\right)\right).$$ (17)

Here Linear_rel_pred_ is a linear transformation layer that includes the bias term and ${\hat{p}}_{t}^{\text{rel}}$ is the predicted distribution of the relation-types.

We used the cross-entropy loss for the training:

$$L_{t,\text{rel}}=-\overset{\left|p_{t}^{\text{rel}}\right|}{\underset{i=1}{\sum }}p_{t,i}^{\text{rel}}\log\left({\hat{p}}_{t,i}^{\mathrm{rel}}\right).$$ (18)

Here $p_{t}^{\text{rel}}$ is one-hot vector, whose dimensions correspond to a relation label defined in Table 2.

### 5.4 Response prediction layer

We add an additional network for guiding the consistent multi-floor dialogue structure. We added this network because responses generated using disentangled dialogue structure are more likely to produce the expected subsequent response than using dialogue history containing entangled miscellaneous information. This layer reconstructs the next turn’s response using the latent variable (in other words, non-explicit latent representations) provided by the hierarchical encoding layer and the information about the current turn’s explicit dialog structure (antecedents and relation-types) provided by the conversation disentanglement layer.

We introduce sequence-to-sequence (seq2seq) model (Vinyals and Le, 2015) for reconstructing the next turn’s response, as follow:

$$h_{t,0}^{\text{seq}2\text{seq}}={\text{Linear}}_{\text{seq}2\text{seq}}\left(\left[p_{t}^{\text{rel}};{\hat{h}}_{t}^{\mathrm{attn}};{{\hat{h}}_{t}}^{\mathrm{ant}}\right]\right),$$ (19)

$$h_{t,i}^{\text{seq}2\text{seq}}={\text{GRU}}_{\text{seq}2\text{seq}}\left(h_{t-1}^{\text{seq}2\text{seq}},\text{Embedding}\left(w_{t+1,i}\right)\right),$$ (20)

$${\hat{p}}_{t,i}^{\text{seq}2\text{seq}}=\text{softmax}\left({\text{Linear}}_{\text{proj}}\left(h_{t,i}^{\text{seq}2\text{seq}}\right)\right).$$ (21)

Here, the initial hidden vector *h*_*t*,0_ that is fed into the seq2seq model is a compressed combination of two types of attention vectors and relational-type prediction result.

We used the cross-entropy loss for the training based on *teacher forcing* technique:

$$L_{t,\text{seq}2\text{seq}}=-\overset{\left|U_{t+1}\right|}{\underset{i=1}{\sum }}\overset{\left|{\hat{p}}_{t,i,j}^{\text{seq}2\text{seq}}\right|}{\underset{j=1}{\sum }}p_{t,i,j}^{\text{seq}2\text{seq}}\log\left({\hat{p}}_{t,i,j}^{\mathrm{seq2seq}}\right).$$ (22)

Here $p_{t,i}^{\text{seq}2\text{seq}}$ is one-hot vector, whose dimensions correspond to a *i*-th word in *U*_*t*+1_.

### 5.5 Objective function

We have to optimize the above three models jointly. In this study, we introduce a multi-task loss, which combines each prediction loss of the antecedent, the relation-type, and the response predictor. In multi-task learning, we interpolate the loss functions of three tasks:

$$L=\frac{1}{N}\overset{N}{\underset{t=1}{\sum }}\left(\gamma_{\text{ant}}L_{t,\text{ant}}+\gamma_{\text{rel}}L_{t,\text{rel}}+\gamma_{\text{seq}2\text{seq}}L_{t,\text{seq}2\text{seq}}\right).$$ (23)

Here *N* is the dialogue length. *γ*_ant_, *γ*_rel_, and *γ*_seq2seq_ are the weights for adjusting the importance of each predictor in the loss calculation.

## 6 Experimental settings

In our experiment, we evaluated the dialogue structure parsing performance of our proposed model, based on an ablation. In this section, we describe the dataset for the training and evaluation, the setting of the model training, and the evaluation metrics.

### 6.1 Dataset

We used a dataset (Traum et al., 2018) that contains dialogues collected at Experiment 1 and Experiment 2. The dialogues were annotated based on a previously described scheme (Traum et al., 2018), which was specifically designed to handle multiple dialogue floors. As shown in Table 3, these dialogue data consist of 48 dialogues (1829 transactions) executed by several different commanders.

**TABLE 3:** Numbers of dialogues, utterances, and transactions.

	Dialogues	Utterances	Transactions
Experiment. 1	24	4527	780
Experiment. 2	24	6994	1049

To evaluate the parsing performance of the proposed model, we randomly divided all of the dialogues in Experiment. 1 and Experiment. 2 into six subsets and applied double cross-validation (Mosier, 1951). We used a single subset for validation and a test-set for each, and the remaining subset was used as training data. We evaluated every possible combination of training, validation, and test-set and the final performance by a majority vote on the prediction results of the models, which share the same test-set.

### 6.2 Model settings

We evaluated the dialogue structure parsing performance of the proposed model in multi-floor dialogues based on an ablation study. Specifically, we evaluated the performance of models trained by a combination of the following five components described in Section 5.

• Floor is the case where the floor information is taken into account to obtain a vector representation of utterances.
• Attn is the case using the supervised-attention in Eqs 9–11 for predicting antecedent utterances.
• Ant is the case using the implicit-attention in Eqs 14, 15.
• Rel is the case of predicting the relation-type of utterances in Eq.17.
• Seq2Seq is the case where each turn predicts the next turn’s response in Eqs 19–21.

Here, if all components are used, they correspond to the full set of the proposed model described in Section 5. If some components are not used, the corresponding parts are completely removed from the network.

We also compared the cases based on both the **Online** and **Offline** models. The proposed model described in Section 5 uses uni-directional GRUs in the context encoder to make predictions for each utterance *U*_*t*_; this means the model only uses previous contexts without subsequent contexts in the prediction for each utterance *U*_*t*_. We call this setting **Online**. The online model is important for real-time dialogue robot processing, which can only use the observed information based on the interaction sequence. In contrast, we also considered a model that uses the whole information in dialogue to make predictions for each utterance *U*_*t*_; this means the model cannot start parsing during the dialogue. We call this setting **Offline**. We built the offline model only using bidirectional-GRUs instead of unidirectional-GRUs in the context encoder. In general, the offline-model has the advantage that information from the entire dialogue is available and can therefore be widely used in situations where progressive parsing is not required.

We used the same hyper-parameter settings in each setting. The vocabulary size was 500, the word embedding size was 100, and the hidden vector size was 300. We used byte pair encoding (BPE) for tokenization (Sennrich et al., 2016). In training, we used a mini-batch size of 32 and an Adam optimizer (Kingma and Ba, 2014) with a learning rate of 1e-3.

To determine the parameters of the loss function, we utilized the Bayesian optimization tool, Optuna. ^3^However, it was found that the case with equal weights performed the best on average. Therefore, we empirically set *γ*_ant_, *γ*_tu_, and *γ*_rel_ to 1. In the relation-type prediction, we integrated the ‘acknowledgement,” “clarification,” and “question-response” sub-types into these classes because some sub-types rarely appeared in the dataset. In addition, we defined an additional relation-type label (#) corresponding to where an utterance has no antecedent (#1 and #6 in Table 1) or its relation-type cannot be identified or is undefined.

### 6.3 Evaluation metrics

We defined the micro and meso-level evaluation metrics for our dialogue structure parsing task. For the micro-level evaluation, we defined the label prediction performances of the antecedents (including no-antecedents), and the relation-types by precision (**Prec.**), recall (**Rec.**), and **F1**. Note that we took the relative position of each utterance from its antecedent as a label to compute the metrics when evaluating the conversation disentanglement (antecedent prediction) performance. If the relative position is zero (#), it means the beginning utterance of the TU. In other words, we compared the difference between the position of predicted antecedents and actual antecedents. We also introduced metrics for the meso-level structure (Traum and Nakatani, 1999) in dialogues to evaluate the consistency of the parsing results. We used the following two metrics:

• GraphAcc is the ratio of the transaction units that perfectly predicted the antecedents for each utterance within the transaction unit.
• GraphAcc w/rel is the ratio of the transaction units that perfectly predicted the antecedents and the relation-types for each utterance within the transaction unit.

Note that the meso-level metrics are stricter than the micro-level metrics, which judge the prediction result of each utterance. Furthermore, we employed Perplexity (**PPL**) to evaluate the performance of the response generation model trained as an auxiliary network for enhancing the performance of dialogue structure parsing. PPL is a measure of how well a language model predicts a given set of data, and a lower perplexity score indicates better performance of the language model in accurately predicting the data (responses according to given dialogue contexts).

## 7 Experimental results

Tables 4, 5 show the performances of each dialogue structure parser corresponding to both online and offline settings. Here, online indicates that the model used only the preceding context of each utterance. Offline indicates that the model used the dialogue entirely for parsing. Majority is a baseline that always picks the most frequent label. $\bar{\text{Prec.}}$ , $\bar{\text{Rec.}}$ , and $\bar{\text{F}1}$ are the weighted averages^4^ of the precision, recall, and F1 scores of the predicted labels. The brackets are the prediction results where the oracle antecedent and relation-type were fed into the model. We conducted paired t-tests to compare the prediction performances (PPL, $\bar{\text{F}1}$ scores, GraphAcc, and GraphAcc w/rel) between the full configuration model and other models on each test set obtained through cross-validation. The significance levels of *p* < 0.01 and 0.5 > *p* ≥ 0.01 were denoted by ** and * symbols, respectively. The asterisks in parentheses show the comparison results between the full configuration model using oracle labels, and other models when using oracle labels (if not available oracle labels, we only used predicted labels).

**TABLE 4:** Prediction performances of multi-floor dialogue structures using online models.

Online models	Seq2Seq	Ant				Rel			
	PPL	$\bar{\text{Prec.}}$	$\bar{\text{Rec.}}$	$\bar{\text{F}1}$	GraphAcc	$\bar{\text{Prec.}}$	$\bar{\text{Rec.}}$	$\bar{\text{F}1}$	GraphAcc w/rel
Majority	-	23.59	48.57	31.76	-	8.54	29.22	13.21	-
Ant + Attn + Rel + Seq2Seq + Floor	3.23	89.23	89.22	89.16	69.10	92.56	93.18	92.79	66.32
	(3.43)					(94.49)	(94.88)	(94.64)	(68.23)
-w/o Ant	3.73*	-	-	-	-	92.52	92.86	92.47	-
	(3.73*)							(**)	
-w/o Attn	3.12	89.18	89.18	89.12	69.10	92.85	93.20	92.86	66.21
	(3.11)					(94.70)	(94.93)	(94.71)	(67.68)
-w/o Rel	3.89**	88.99	89.00	88.90*	68.01*	-	-	-	-
	(3.85**)								
-w/o Seq2Seq	-	88.99	88.99	88.90	68.45	92.43	92.96	92.58	65.11
						(94.63)	(94.95)	(94.62)	(67.41)
-w/o Floor	5.62**	86.24	86.32	84.73**	54.94**	89.00	90.49	89.41**	50.79**
	(5.62**)					(91.51)	(92.97)	(91.99**)	(52.97**)
Ant + Attn + Floor	-	88.94	88.92	88.80	67.74	-	-	-	-
								-	-
Rel + Attn + Floor	-	-	-	-	-	92.53	92.69	92.66	-
								(**)	
Seq2Seq + Attn + Floor	3.32	-	-	-	-	-	-	-	-

**TABLE 5:** Prediction performances of multi-floor dialogue structures using offline models.

Offline models	Seq2Seq	Ant				Rel			
	PPL	$\bar{\text{Prec.}}$	$\bar{\text{Rec.}}$	$\bar{\text{F}1}$	GraphAcc	$\bar{\text{Prec.}}$	$\bar{\text{Rec.}}$	$\bar{\text{F}1}$	GraphAcc w/rel
Majority	-	23.59	48.57	31.76	-	8.54	29.22	13.21	-
Ant + Attn + Rel + Seq2Seq + Floor	3.19	89.72	89.71	89.64	70.92	92.74	93.18	92.85	67.08
	(3.19)					(94.84)	(95.11)	(94.88)	(69.05)
-w/o Ant	3.51*	-	-	-	-	92.52	92.95	92.65	-
	(3.51)							(**)	
-w/o Attn	3.06	89.81	89.75	89.67	70.69	92.72	93.06	92.77	67.08
	(3.06)					(94.97)	(95.09)	(94.88)	(69.98)
-w/o Rel	3.54**	89.45	89.41	89.29*	69.27*	-	-	-	-
	(3.54**)								
-w/o Seq2Seq	-	89.81	89.83	89.74	70.42	92.90	93.38	93.06	66.37
						(94.80)	(95.04)	(94.82)	(68.83)
-w/o Floor	5.27**	88.82	88.73	88.67**	68.45**	90.27	91.29	90.50**	63.47*
	(5.27**)					(92.59)	(93.33)	(92.63**)	(66.70*)
Ant + Attn + Floor	-	89.62	89.60	89.48	70.53	-	-	-	-
Rel + Attn + Floor	-	-	-	-	-	92.43	92.93	92.64	-
								(**)	
Seq2Seq + Attn + Floor	3.11	-	-	-	-	-	-	-	-

The results showed that dialogue structure parsing performance (conversation disentanglement and relation-types prediction) of offline models has improved from the online models. This result indicates that subsequent contexts help label prediction for each utterance, but we can have enough prediction accuracy even if the model does not have the subsequent context (online setting). This may be due to the simplicity of the dialogue task addressed in this study, which consists of a simple set of utterances, commands, and robot actions. Additionally, it may be because past context is crucial for resolving dialogue dependencies, while subsequent context is only supplementary in the setting of this study. Although this study assumed three dialogue participants, we can expect more complex turn-taking and information exchange when the number of participants is larger, or the number of floors is increased. In such cases, the importance of subsequent context is likely to increase, and the advantage of using offline models is expected to be even greater. When the multi-task models use oracle antecedents to predict the relation-type, we can further improve the performance of dialogue structure parsing. In addition, the multi-task model with multiple different prediction modules showed better overall performance than the single-task model, which predicts only antecedents and relation-types.

The ablation study showed the highest dialogue structure parsing performance when we used the full-configuration models and the full-configuration models without the Attn module (implicit-attention). Although implicit-attention did not contribute to the relation-type prediction performance, it can be seen that supervised-attention (conversation disentanglement model) slightly contributes to the prediction performance of the relation-type. In other words, it is better for us to explicitly teach them what the network should pay attention rather than for them to learn it themselves. In contrast, when the models did not predict the relation-type, the performance of conversation disentanglement was significantly decreased. This supports our hypothesis that multi-task learning of conversation disentanglement and relation-type prediction improves the performance of dialogue structure parsing.

Regarding when information about the floor to which an utterance belongs is not available (w/o Floor), the performance of the dialogue structure parsing model is found to be significantly decreased. This result suggests that a dialogue structure parsing model that does not focus on explicit floor information will not work well in our setting. However, our model assumed that the configuration of floors (occurrence and disappearance of floors and participants of floors) in a multi-floor dialogue is static, but challenges remain where the floor configuration is dynamic.

Regarding the response prediction model (seq2seq), we found that connecting it to the end layer of the dialogue structure parsing model slightly improved the overall performance of the dialogue structure parsing. This supports our hypothesis that learning a response prediction task based on the implicit and explicit dialogue structure representations predicted by the dialogue structure parsing model is useful as an auxiliary objective function to improve the performance of the dialogue structure parsing model. However, the response generation performances (PPL) of seq2seq models in our multi-task models is comparable to the seq2seq models learned without considering the dialogue structure parsing results. However, this is only a comparison of multi-task models, in which the models are trained to include other dialogue structure parsing tasks, *versus* a single-task model that optimizes the PPL directly and is not a fair comparison. When discussing our dialogue structure parsing models from a dialogue system perspective, PPL is comparable to the single-task models. However, our models have an advantage that can explain the rationale for response generation by the dialogue structure parsing results. Note that in this experiment, the loss weights corresponding to each task in training the model were set equally to 1, but a further promising outcome may be obtained by dynamically adjusting these loss weights during the training process.

Table 6 shows the results of the antecedent prediction in the full-configured online model (Ant + Attn + Rel + Seq2Seq + Floor). Here each label indicates the relative position from each utterance to its antecedent. Note that this table only shows the prediction results by considering a maximum of ten previous utterances. We merged a few cases when the antecedent of the utterance is not included in the ten previous utterances or the utterance has no antecedent, into zero label (#). Our model can predict antecedents with high performance when the relative position was not distant. On the other hand, the prediction performance was below 80% when the relative positions were distant (greater than five in absolute). This result suggests the difficulty of addressing long-term dependency in dialogues.

**TABLE 6:** Antecedent prediction performance of full-configured online model.

Position	Precision	Recall	F1	Count
−10	34.88	71.43	46.88	21
−9	60.71	51.52	55.74	33
−8	77.50	59.62	67.39	52
−7	80.77	74.12	75.31	85
−6	91.52	84.83	88.05	178
−5	75.24	58.52	65.83	135
−4	85.93	83.23	84.56	477
−3	88.61	85.92	87.25	1023
−2	91.43	91.47	91.45	2509
−1	89.93	94.53	92.18	4262
0 (#)	88.77	84.67	86.67	2746

Utterances in a multi-floor dialogue can be classified into three types: starting a new TU (Start), and continuing the currently open TU (Continue), resuming another TU that is already open (Other). Table 7 shows the prediction performance of the antecedent prediction corresponding to such three statuses of TUs (Kawano et al., 2021). The results showed that the model suffered in the case of predicting the utterance of another TU already open as the antecedent compared to other cases. This suggests that dialogue structure parsing performance critically degrades when attempting to handle more complex dialogues tasks with multiple transactions in parallel. Thus further improvements are needed for conversation disentanglement.

**TABLE 7:** Antecedent prediction performance corresponding to types of TUs by full-configured online model.

Tu-label	$\bar{\text{Prec.}}$	$\bar{\text{Rec.}}$	$\bar{\text{F}1}$	Count
Start	97.54	94.53	95.74	1829
Continue	88.35	88.45	88.35	8599
Other	89.53	86.37	87.70	1093

Table 8 shows the results of the relation-type predictions of the full-configured online model. Here, No-relation (#) is a label corresponding to where utterance has no antecedent or its relation-type cannot be identified or is undefined. Our model showed higher F1 scores in frequent relation-types. There is still a challenge in predicting low-frequent relation-types due to the lack of training data. Ongoing annotation work (Traum et al., 2018) with additional data may remedy this problem. We also need to look at ways to deal with these unbalanced labels.

**TABLE 8:** Relation-type prediction performance of full-configured online model.

Relation-type	Precision	Recall	F1	Count
Expansions				
-continue	86.51	86.60	86.55	955
-link-next	99.37	99.37	99.37	318
-correction	37.50	8.33	13.64	36
-summarization	0.00	0.00	0.00	13
Responses				
-acknowledgement	96.71	96.17	96.44	3366
-clarification	76.40	80.95	78.61	420
-processing	98.73	100.00	99.36	233
-question-answer	66.43	55.23	60.32	172
-other	37.50	9.09	14.63	33
-3rd-turn-feedback	0.00	0.00	0.00	25
-reciprocal-response	0.00	0.00	0.00	5
Translations				
-l	95.64	98.34	96.97	1563
-r	98.30	98.40	98.35	1942
-comment	98.24	97.73	97.99	21
No-relation (#)	89.50	92.64	91.04	2419

Our model decided antecedent labels with the highest prediction probability for each utterance; however, we did not consider the consistency of the prediction results in the sequence of dialogue. To solve this problem, we can introduce a model that takes into account information about the entire prediction results, such as Conditional Random Field (CRF) (Lafferty et al., 2001) for further improvements. In addition, our model ignores the consistency of the graph structure associated with the predicted antecedents and relation-types at inference step. The search for dialogue structures using dynamic programming probably has the potential to improve the performance of our model.

Finally, in Table 9, we show an example of dialogue structure parsing result by our full-configured online model on a fragment of multi-floor dialogue. We displayed the correct labels in brackets when the label was incorrectly predicted, and “#” corresponds to cases where the utterance does not have the antecedent. The first example shows that the model accurately predicts all the antecedents, and relation-types, even if transactions were interleaved. However, the second example includes error predictions. In this example, there are only two TUs, but the model has determined that the utterance has three TUs. The utterance #8 is actually part of the transaction started by the utterance #5. The error will be extended beyond one utterance to multiple utterances when such confusion occurs. In many cases, delays in communication, and differences in the quality of annotations between Experiment.1 and 2 will lead to confusing predictions. Model training and annotation considering information such as the robot’s position, vision, and their timestamps, may be necessary for further improvement.

**TABLE 9:** Examples of the dialogue structure parsing on multi-floor dialogue.

	Left floor		Right floor		Prediction	
#	Commander	DM → commander	DM → RN	RN	Ant	Rel
1	turn right twenty degrees				#	#
2			turn right 20		1	translation-r
3		executing …			1	response-ack
4			image		1	translation-r
5				done image sent	4	response-ack
6	go forward fifteen feet				#	#
7		sent			5	translation-1
8	and go through door on right				6	expansion-cont
9			move forward about 15 feet, going through door on right, image		8	translation-r
10		executing …			8	response-ack
	Left floor		Right floor		Prediction	
#	Commander	DM → commander	DM → RN	RN	Ant	Rel
1	take a picture				#	#
2			image		1	translation-r
3				image sent	2	response-ack
4		sent			3	translation-l
5	turn left ninety degrees				#	#
6			turn left 90		5	translation-r
7		executing …			5	response-ack
8	take a picture after each command				#	#
					(5)	(expansion-cont.)
9				done	6	response-ack
10			take pic after each command		8	translation-r
11			image		8	translation-r
12				image sent	11	response-ack
13		sent			12	translation-l

## 8 Conclusion

We built a neural dialogue structure parser with an attention mechanism that applies multi-task learning to automatically identify the dialogue structure of multi-floor dialogues. The experimental results showed that our proposed model improved the overall dialogue structure parsing performance compared to models trained on single task settings. However, problems remain with the performance of the dialogue structure identification due to the lack of training data, especially for rare labels. We will consider pre-training and the transfer learning of models using still unlabeled dialogue data and existing discourse-relation datasets to prevent this problem. To improve the consistency of predicted dialogue structure, we also explore the possibility of introducing powerful models or inference strategies of tasks related to predicting graph structure in a document, such as a dependency parsing (Nivre, 2010) and discourse parsing based on rhetorical structure theory (Mann and Thompson, 1988; Webber et al., 2012).

This study has developed the first baseline model for automatic identification of dialogue structure on multi-floor dialogues. It has the potential for applying to the automatic annotation of dialogue structure on multi-floor dialogues for robot operation and encourages the development of a dialogue manager and robot navigator in multi-floor settings. However, our model deals with a rather artificial multi-floor dialogue structure analysis task, and it remains to be seen whether it can be applied to multi-floor dialogue structure analysis tasks in other domains or with different floor structures. Therefore, constructing a new multi-floor dialogue dataset following the setup of practical multi-party dialogue datasets (Janin et al., 2003; Uthus and Aha, 2013) used in existing studies would be a promising direction to demonstrate the usefulness of our model.

## Data Availability

The data analyzed in this study is subject to the following licenses/restrictions: The original dialogue data used in this paper was distributed by the United States Army Research Laboratory and required a contract with them to use it. Requests going forward to access these datasets should be directed to Claire Bonial (claire.n.bonial.civ@army.mil) or Stephanie Lukin (stephanie.m.lukin.civ@army.mil).

## Footnotes

1 This scheme was later extended in (Bonial, 2021), however we used the version from (Traum et al., 2018).

2 Here, the hierarchical encoder can also be constructed using the transformer model. However, in this paper, the transformer model was not stable due to the size of the dataset available for training, thus, an RNN-based model was adopted.

3
https://github.com/optuna/optuna.

4 We calculated the weighted averages based on the label frequencies.

## Author disclaimer

The views and conclusions contained in this document are those of the authors and should not be interpreted as representing the official policies, either expressed or implied, of the Army Research Office or the United States Government. The United States Government is authorized to reproduce and distribute reprints for Government purposes notwithstanding any copyright notation herein.
